# Current Status of Antimicrobial Resistance in Taiwan

**DOI:** 10.3201/eid0802.010244

**Published:** 2002-02

**Authors:** Po-Ren Hsueh, Cheng-Yi Liu, Kwen-Tay Luh

**Affiliations:** * National Taiwan University Hospital, National Taiwan University College of Medicine, Taipei, Taiwan; † Taipei Veterans General Hospital and National Yang-Ming University, Taipei, Taiwan

**Keywords:** antimicrobial resistance, Taiwan

## Abstract

While some trends in antimicrobial resistance rates are universal, others appear to be unique for specific regions. In Taiwan, the strikingly high prevalence of resistance to macrolides and streptogramin in clinical isolates of gram-positive bacteria correlates with the widespread use of these agents in the medical and farming communities, respectively. The relatively low rate of enterococci that are resistant to glycopeptide does not parallel the high use of glycopeptides and extended-spectrum beta-lactams in hospitals. The evolving problem of extended-spectrum beta-lactamase-producing *Escherichia coli* and *Klebsiella pneumoniae* isolates is substantial, and some unique enzymes have been found. Recently, some gram-negative bacteria (e.g., *Pseudomonas aeruginosa* and *Acinetobacter baumannii)* that are resistant to all available antimicrobial agents including carbapenems have emerged.

Antimicrobial resistance has become a major health problem worldwide, affecting every country to some degree. It is an inevitable consequence of the inappropriate use of antibiotics in humans and animals. In Europe and North America, methicillin-resistant *Staphylococcus aureus* (MRSA), penicillin-nonsusceptible *Streptococcus pneumoniae* (PNSSP), vancomycin-resistant enterococci (VRE), and extended-spectrum beta-lactamase (ESBL)-producing E*nterobacteriaceae* have emerged and spread into communities and hospitals. In Taiwan, the widespread use of antimicrobial agents in primary care clinics and animal husbandry has allowed the rapid emergence of resistant bacteria. During the last 2 decades, many antimicrobial agents--such as extended-spectrum cephalosporins, carbapenems, fluoroquinolones, and aminoglycosides--have been introduced and empirically used as first-line drugs to treat these resistant bacteria (*1**,**2*). This has further accelerated the development and dissemination of drug-resistant bacteria. Previous studies in Taiwan have clearly demonstrated the remarkably high prevalence of some critically resistant bacteria, such as MRSA, PNSSP, and macrolide-resistant streptococci (*1**,**2*). In addition, several multidrug-resistant bacteria, including ones resistant to carbapenems and fluoroquinolones and pan-drug-resistant gram-negative bacilli, have been isolated from different hospitals (*3**–**6*).

## Approval of Antibiotics

Table 1 shows the years that selected antibiotics were approved in Taiwan. These antibiotics are now widely used to treat various infections, including community-acquired and nosocomial infections. Until now, two gylcopeptides (vancomycin and teicoplanin), two carbapenems (imipenem and meropenem), four macrolides (erythromycin, roxithromycin, clarithromycin, and azithromycin), and six quinolones (nalidixic acid, norfloxacin, ofloxacin, lomefloxacin, ciprofloxacin, and levofloxacin) have been available for clinical use in Taiwan. Most of these drugs were also readily available at drugstores without prescription before 1995.

**Table 1:** Year of approval of selected antimicrobial agents in Taiwan

Antimicrobial agent	Year of approval
Erythromycin	1968
Oxacillin	1970
Gentamicin	1981
Cefotaxime	1983
Amikacin	1986
Ceftazidime	1988
Imipenem	1988
Vancomycin	1983
Ciprofloxacin	1990
Cefepime	1997

## Drug-Resistant Bacteria

The following drug-resistance data were collected from a nationwide resistance survey (Surveillance from Multicenter Antimicrobial Resistance in Taiwan ) of clinical isolates (including those recovered from hospitals and outpatients) from 12 major hospitals as well as isolates causing nosocomial infections from National Taiwan University Hospital (NTUH) in 2000 in Taiwan. These hospitals are located in different parts of the country. The number of beds in these hospitals ranged from 800 to 3,200. All these data were derived by using the disk-diffusion method (*7*). 

Some dilution antimicrobial susceptibility and epidemiology studies, including ≥100 strains published in English-language journals from January 1995 through 2001, were also included. Rather than provide a comprehensive review of all resistance problems in Taiwan, our aim was to point out some of the more critical resistance problems threatening the treatment of infections caused by *Staphylococcus* species, *S. pyogenes*, *Streptococcus pneumoniae*, *Enterococcus* species, and *Mycobacterium tuberculosis* among the gram-positive pathogens, and *Haemophilus influenzae*, *Escherichia coli, Klebsiella pneumoniae*, *Enterobacter* species, *Salmonella* species, *Campylobacter* species, *Pseudomonas aeruginosa*, and *Acinetobacter baumannii* among the gram-negative pathogens. Resistance rates included in this review reflect both intermediate and fully resistant population. Table 2 summarizes the prevalence of antimicrobial resistance among clinical isolates (12 hospitals, including NTUH) and nosocomial isolates (from NTUH only) of some selected bacterial species. The ranges in numbers of clinical isolates of select bacteria (Table 2) recovered from these hospitals were as follows: *Staphylococcus aureus*, 1,889 to 7,516 isolates; beta-hemolytic streptococci, 335 to 1,102; *S. pneumoniae*, 138 to 461; enterococci, 509 to 3,676; *H. influenzae*, 427 to 602; *E. coli*, 1,734 to 9,553; *K. pneumoniae*, 950 to 3,226; *E. cloacae*, 427 to 1,426; nontyphoid *Salmonella*, 94 to 626; *P. aeruginosa*, 1,741 to 4,896; and *A. baumannii*, 896 to 2,434.

**Table 2:** Prevalence of antimicrobial resistance in selected bacteria (all clinical isolates) isolated from 12 major hospitals, including National Taiwan University Hospital (NTUH), in Taiwan in 2000 and in all clinical isolates and isolates causing nosocomial infections from NTUH in 2000^a^

Resistant pathogen	% of isolates	
	2,000 (12 hospitals) (clinical)	2,000 (NTUH) (clinical/nosocomial)
Methicillin-resistant *Staphylococcus* *aureus*	53-83	65/74
Erythromycin-resistant beta-hemolytic streptococci	30-51	34/-
Penicillin-nonsusceptible *Streptococcus pneumoniae*	60-84	77/-
Erythromycin-resistant *S. pneumoniae*	67-100	89/-
Gentamicin-resistant (high-level) enterococci	36-54	48/54
Vancomycin-resistant enterococci	1-3	3/2
Ampicillin-resistant *H. influenzae*	45-73	61/-
Cefotaxime-resistant *Escherichia coli*	5-19	12/19
Ciprofloxacin-resistant *E. coli*	11-33	20/29
Cefotaxime-resistant *Klebsiella pneumoniae*	4-34	9/18
Ciprofloxacin-resistant *K. pneumoniae*	5-33	9/16
Cefotaxime-resistant *E. cloacae*	36-68	45/49
Ampicillin-resistant non-typhoid *Salmonella*	44-69	56/-
Cefotaxime-resistant non- typhoid *Salmonella*	1-4	2/-
Quinolone resistant non-typhi *Salmonella*	0-16	0/-
Ceftazidime-resistant *Pseudomonas aeruginosa*	4-21	13/10
Imipenem-resistant *P. aeruginosa*	3-16	14/10
Ciprofloxacin-resistant *P. aeruginosa*	10-36	15/10
Imipenem-resistant *Acinetobacter baumannii*	0-19	19/16
Ciprofloxacin-resistant *A. baumannii*	54-74	54/42

^a^Susceptibility of these bacteria was determined by the standard disk-diffusion method.

## Gram-Positive Bacteria

### MRSA

MRSA was first documented in Taiwan in the early 1980s (*8*). Since then, there has been a remarkable increase in prevalence of MRSA in nosocomial infections (from 26.7% in 1990 to 75% to 84% in 1998-2000) (*9*). Several dominant clones have been documented in hospitals (*9*). The prevalence of MRSA in community-acquired infections remains unclear, although the incidence of MRSA among patients of outpatient departments is estimated to be 40% (*1*). Data from a survey of >5,000 clinical isolates of *S. aureus* at the NTUH from January 1999 to June 2001 using brain-heart-infusion agar plus 4 mg/L of vancomycin showed results negative for vancomycin-intermediate or -resistant strains.

## PNSSP and Multidrug-Resistant *Streptococcus pneumoniae* (MDRSP)

The overall prevalence of clinical isolates of PNSSP in 1999-2000 was 60% to 80%, including 20% to 30% penicillin-intermediate- and 40% to 50% penicillin-resistant strains (*10**–**16*). This prevalence of PNSSP was slightly lower than that in Korea and higher than that in most other geographic areas (*15**,**16*). All PNSSP were resistant to multiple antibiotics (*13**,**16*). This resistance was higher among nasopharyngeal isolates from children (*12*). Approximately 60% of the PNNSP isolates were also not susceptible to extended-spectrum cephalosporins and carbapenems (*13*). Most of these PNSSP belong to serotypes 23F, 19F, 6B, and 14 (*13**,**15*). Wide dissemination of multiple high-level penicillin-, extended-spectrum cephalosporin-, and macrolide-resistant clones as well as the Spain 23F clone contributes to the high rates of resistance to these drugs in clinical isolates of *S. pneumoniae* (*14**,**17*). Only one clinical isolate was reported to be resistant to fluoroquinolones (*18*).

## VRE and Glycopeptide-Resistant Staphylococci

The first clinical isolate of Van-A-phenotype VRE (*E. faecalis*) was found in 1995 (*19*). Since then, isolation of VRE remains rare and accounts for <3% of all clinical isolates of enterococci (*20**,**21*). The proportion of Enterococcus hospital isolates resistant to vancomycin in Taiwan is low compared with those in North America and Europe (*22*), a finding that needs further investigation. However, an increase in VRE isolation associated with the continuous widespread use of glycopeptides in a Taiwanese university hospital was observed (*23*). Furthermore, interhospital and nosocomial spread of some VRE clones, particularly one *vanB2* *E. faecium* clone, or long-term persistence of multiple clones in hospitalized patients still exists (*21**,**24*). Although avoparcin has been approved for veterinary use since 1977, this agent has been banned in the farming industry since 2000 (*24*). Glycopeptide resistance has been found in some isolates of coagulase-negative staphylococci, particularly in *S. simulans* and *S. warneri* (*25*).

## Macrolide-Resistant Streptococci

Under the increasing and highly selective pressure of macrolide usage in Taiwan, the prevalence of macrolide resistance and distribution of M-phenotype (*mef* gene-positive) among macrolide-resistant isolates vary among different streptococcal species (Figure) (*26**–**31*). More than 90% of the *S. pneumoniae* isolates were resistant to macrolides, and approximately two thirds exhibited high-level resistance (MLS_B_ phenotype-*erm* gene-positive) (*29*). However, macrolide resistance accounted for 50% to 60% of all clinical isolates of *S. pyogenes,* and a stepwise increase of proportion of M phenotype was clearly demonstrated (*29*). 

**Figure:**
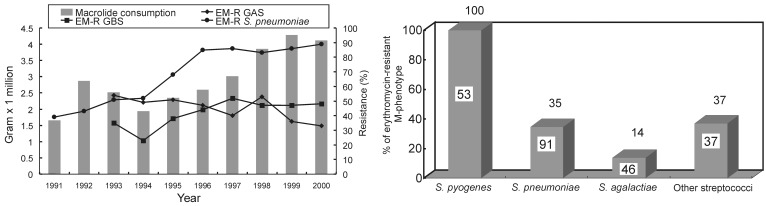
A, Macrolides consumption (gram x 1000,000) in Taiwan and the trends of erythromycin-resistant group A *Streptococcus* (EM-R GAS), group B *Streptococcus* (EM-R GBS), and *S. pneumoniae* in National Taiwan University Hospital from 1991 to 2000*.* Macrolides include intravenous and oral forms of erythromycin and oral forms of clarithromycin, roxithromycin, and azithromycin. B,. Distribution of erythromycin-resistant M-phenotype among isolates of streptococci. Other streptococci include Groups C, F, and G, and viridans group streptococci. Number in each bar indicates the percentage of erythromycin-resistant isolates. Number above each bar indicates the percentage of M-phenotype among erythromycin-resistant isolates.

## Streptogramin-Resistant Gram-Positive Cocci

Quinupristin-dalfopristin is not available for clinical use in Taiwan; nevertheless, the incidence of resistance to this agent was high (51%) in vancomycin-resistant *E. faecium* (*25*). Three resistant *E. faecium* isolates were recovered from animal sources (pigs) in Taiwan. Restricted use of virginiamycin, which has been widely used in animal feed for >20 years in this country, might be required to alleviate quinupristin-dalfopristin resistance among bacteria from human sources (*25*).

## Multidrug-Resistant *Mycobacterium tuberculosis* (MDRTB)

The prevalence of pulmonary tuberculosis (TB) in adults was 0.65% in 1993 and the associated death rate was 6.93 per 100,000 in 1998 (*32*). The overall incidence of isoniazid-resistant *M. tuberculosis* was 31.5%. The incidence of primary resistance (isolates from patients with newly diagnosed TB who had no prior history of anti-TB therapy or from patients whose anti-TB therapy was begun <2 weeks) was 12.0%; the incidence of acquired resistance (isolates from patients who had a prior history of anti-TB medication) was 63.0%. The overall incidence of MDRTB was 17.3% (primary resistance 1.6%; acquired resistance 46%) (*33*). An aggressive intervention program, such as expanded use of directly observed therapy, short course, is ongoing to improve the cure rate of TB and to decrease the resistance rate.

## Gram-Negative Bacilli

### *H. influenzae* and *Moraxella catarrhalis*

The annual incidence of invasive *H. influenzae* type b disease in children <5 years old was 1.6 to 1.9 per 100,000 population per year before the introduction of conjugated Hib vaccine in 1995 (*34*). Beta-lactamase production was found in 50% to 60% of *H. influenzae* and in >95% of *M. catarrhalis.* BRO-1 isoform accounts for 88% of all beta-lactamase producers of *M. catarrhalis* (*16**,**35**,**36*). Among amoxicillin-resistant *H. influenzae* isolates, beta-lactamase nonproducers were rare (<2%) (*16*). A continuing upsurge of *H. influenzae* isolates resistant to macrolide (30%) and to trimethoprim-sulfamethoxazole (50%) during the last decade has become evident (*16**,**35*).

### Enterobacteriaceae

The proportion of isolates of *K. pneumoniae* exhibiting the ESBL phenotype has increased progressively from 3.4% in 1993 to 10.3% in 1997 in NTUH (*37*). Approximately one fifth of the ESBL-producing *K. pneumoniae* were also resistant to ciprofloxacin (*37*). From 1998 through 2000, several reports from different hospitals showed that ESBL production accounts for 8% to 30% of clinical isolates of *K. pneumoniae.* .Those producing SHV-5 and SHV-12 predominated. In addition, four novel beta-lactamases (CMY-8, SHV-25, SHV-26, and IMP-8) were identified in 2000 in Taiwan (*38**–**42*). Among the ESBL-producing *E. coli* isolates, which accounted for 1.6% to 6.7%, strains having CTX-M-3 and CMY-M-2 were disseminated in Taiwan (*39**,**43*). In Taiwan, the previous belief that characteristically susceptible strains (uniformly susceptible to cephalosporins) of *K. pneumoniae* caused primary liver abscess, an endemic disease entity in patients with diabetes mellitus, has now been disproved because two cephalosporin-resistant *K. pneumoniae* strains causing primary liver abscess have been found (*44**–**46*).

More than 40% of clinical isolates of nontyphoid *Salmonella* species were resistant to multiple antibiotics (ampicillin, chloramphenicol, and trimethoprim-sulfamethoxazole). Resistance to cefotaxime and fluoroquinolones was estimated to be low (1% to 3%) (*47*).

### *P. aeruginosa*, *A. baumannii*, and Other Bacteria

*P. aeruginosa, A. baumannii*, and other nonfermentative gram-negative bacilli are usually resistant to various antimicrobial agents. A high proportion of clinical isolates, particularly those recovered from patients in intensive-care units, that are resistant to some last-line agents (ceftazidime, amikacin, ciprofloxacin, and carbapenems) have now been found in Taiwan (*3**–**6**,**48**,**49*). A small outbreak of infections (three patients) caused by a pandrug-resistant *P. aeruginosa* (serogroup O:4) clone in an intensive-care burn unit from April 1997 to May 1997 has been identified (*3*) This clone had been isolated from a patient on the same unit 5 months before the outbreak (*3*). Among *P. aeruginosa* isolates with reduced susceptibilities to imipenem, VIM-2 and VIM-3 are the predominant metallo-beta-lactamases (*50*). Furthermore, clonal dissemination of VIM-3-producing *P. aeruginosa* has been found among hospitals in Taiwan (*50*). Strains of ceftazidime- and ciprofloxacin-resistant *A. baumannii* causing severe community-acquired pneumonia have emerged (*49*). Infections caused by *Chryseobacterium indologenes*, a multidrug-resistant nosocomial pathogen, appear to be another emerging problem in Taiwan (*5*). Isolates of the *Chryseobacterium* genus have remarkable discrepancies of susceptibility results by the disk-diffusion and dilution method. Vancomycin is not recommended as a drug of choice for treating *C. meningosepticum* meningitis or other infections caused by *Chryseobacterium* species because these isolates are highly resistant to vancomycin when the standard agar dilution method is used (*4*).

Several multidrug-resistant (extended-spectrum cephalosporins, ciprofloxacin, or carbapenem resistance) *Aeromonas* species have been reported (*51**,**52*). A derepressed mutant of *A. hydrophila,* which overexpresses beta-lactamases and shows resistance to extended-spectrum cephalosporins, is used if treatment with cefotaxime for *Aeromonas* bacteremia fails (*52*). High prevalence of ciprofloxacin resistance for human isolates of *Campylobacter jejuni* (52%) and *C. coli* (75%) may be attributable to the widespread use of quinolones in poultry in Taiwan (*53**,**54*).

## Strategy for Resistance Control in the 21st Century

By the end of the 20th century, many measures to control resistance problems had been instituted in Taiwan. Antibiotics had been removed from the list of available nonprescription drugs at drugstores. Antibiotic interventions had been implemented in many hospitals, particularly in intensive-care units, to alleviate the high prevalence of resistance among nosocomial pathogens. In 2000, the Council of Agriculture in Taiwan prohibited the use of several antimicrobial agents (such as avoparcin, kanamycin, kitasamycin, lasalocid, spiramycin, salinomycin, and streptomycin), which had been widely used as growth promoters or prophylactic agents in animal husbandry in Taiwan during the past 2 to 3 decades, because they may select for critical forms of resistance in human pathogens in food-producing animals (*54*). Further research is ongoing to reduce the risk for increasing resistance in human pathogens caused by antibiotic use in animal husbandry. In the new millennium, the Center for Disease Control, Department of Health, in Taiwan, has made control of antimicrobial resistance a major goal. The two main tasks are to restrict use of antibiotics for trivial upper respiratory tract infections and to avoid inappropriate use of antibiotics for surgical prophylaxis.
